# Users’ and therapists’ perceptions of myoelectric multi-function upper limb prostheses with conventional and pattern recognition control

**DOI:** 10.1371/journal.pone.0220899

**Published:** 2019-08-29

**Authors:** Andreas W. Franzke, Morten B. Kristoffersen, Raoul M. Bongers, Alessio Murgia, Barbara Pobatschnig, Fabian Unglaube, Corry K. van der Sluis

**Affiliations:** 1 University of Groningen, University Medical Center Groningen, Department of Rehabilitation Medicine, Groningen, The Netherlands; 2 University of Groningen, University Medical Center Groningen, Center for Human Movement Sciences, Groningen, The Netherlands; 3 Gait and Motion Analysis Laboratory, Orthopedic Hospital Speising, Vienna, Austria; Manchester Metropolitan University - Cheshire Campus, UNITED KINGDOM

## Abstract

**Objective:**

To describe users’ and therapists’ opinions on multi-function myoelectric upper limb prostheses with conventional control and pattern recognition control.

**Design:**

Qualitative interview study.

**Settings:**

Two rehabilitation institutions in the Netherlands and one in Austria.

**Subjects:**

The study cohort consisted of 15 prosthesis users (13 males, mean age: 43.7 years, average experience with multi-function prosthesis: 3.15 years) and seven therapists (one male, mean age: 44.1 years, average experience with multi-function prostheses: 6.6 years). Four of these users and one therapist had experience with pattern recognition control.

**Method:**

This study consisted of semi-structured interviews. The participants were interviewed at their rehabilitation centres or at home by telephone. The thematic framework approach was used for analysis.

**Results:**

The themes emerging from prosthesis users and therapists were largely congruent and resulted in one thematic framework with three main themes: control, prosthesis, and activities. The participants mostly addressed (dis-) satisfaction with the control type and the prosthesis itself and described the way they used their prostheses in daily tasks.

**Conclusion:**

Prosthesis users and therapists described multi-function upper limb prostheses as more functional devices than conventional one-degree-of-freedom prostheses. Nonetheless, the prostheses were seldom used to actively grasp and manipulate objects. Moreover, the participants clearly expressed their dissatisfaction with the mechanical robustness of the devices and with the process of switching prosthesis function under conventional control. Pattern recognition was appreciated as an intuitive control that facilitated fast switching between prosthesis functions, but was reported to be too unreliable for daily use and require extensive training.

## Introduction

Dissatisfaction with myoelectric hand prostheses has been reported to cause rejection rates as high as 23% and these devices have not gained widespread use despite this technology being available for more than 40 years.[1] In attempts to improve acceptability and usability, increasingly sophisticated prosthetic hands and control methods have been introduced.[2–4] As a result, several companies now offer multi-articulated prosthetic hands with a variety of functions. Moreover, the first commercial control system based on pattern recognition algorithms (CoApt COMPLETE CONTROL)[5] was introduced to the market in 2014. It aims to offer faster and more intuitive control than conventional methods.

These developments appear to match prosthesis users’ needs. User surveys reported that there was a desire for more functions for prosthetic hands (such as greater degrees of freedom in the wrist and multiple grip types) and more dexterous control.[1,6–12] However, while both multi-function prosthetic hands as well as pattern recognition control methods were made available for prosthesis users in the last decade, opinions on these technologies from the perspective of users or healthcare professionals have seldom been addressed in the scientific literature.

The impact of multi-function prosthetic hands was investigated in two case studies and a longitudinal crossover study of six prosthesis users.[10,13,14] They presented preliminary evidence that users preferred multi-function prostheses over their standard prosthetic hands, even though functional scores were not consistently higher with multi-function devices. Specifically, users reported that the variety of grip modes and multi-articulating prosthetic fingers facilitated easier grasping and a more reliable grip on objects. However, given the small number of studies and participants, more data are needed to support these conclusions. Importantly, these studies included only participants who used conventional control methods, which raises the question of how users perceive multi-function prosthetic hands under pattern recognition based control, since this control type has only recently been made available.

Pattern recognition control has gained considerable attention in the scientific literature, but only a few studies have compared this control to conventional control methods in prosthesis users and even fewer studies have investigated users’ opinions on pattern recognition control. Two recent publications compared pattern recognition and conventional control in three and eight individuals with amputations, respectively.[15,16] They found that two out of three users and seven out of eight users preferred pattern recognition to conventional control. The main reason was that with pattern recognition control, users could make seamless transitions between prosthesis functions (e.g., switching from wrist rotation to hand open/close), whereas conventional control required users to perform specific “mode switching” muscle contractions (e.g., co-contraction of agonist-antagonist muscle pairs).

In contrast to these findings, a case study found no significant difference in functional scores and satisfaction when comparing pattern recognition and conventional control.[17] Furthermore, a qualitative study in which the subjects completed home trials with a pattern recognition system reported that the majority of the users preferred their personal prostheses over a pattern recognition controlled system.[18]

In summary, only a few publications have investigated the benefits and impact of multi-function prostheses and pattern recognition control from the perspective of users or healthcare professionals. Moreover, only two of the studies collected and analysed qualitative data.[10,18] Finally, the findings were not always conclusive and opinions on these technologies have rarely been described in great detail.

The aim of this study was to fill in this knowledge gap by providing a qualitative approach to the question of how users and therapists experience multi-function prostheses with conventional or pattern recognition control.

## Methods

This study was conducted by (1) the Department of Rehabilitation Medicine, University Medical Centre Groningen, the Netherlands, and (2) Orthopaedic Hospital Speising, Vienna, Austria. The Medical Ethical Committee of the University Medical Centre Groningen concluded that this study did not fall under the scope of medical-scientific research, so no formal ethical approval was required in the Netherlands (METc 2016/288). The Ethics Commission of Vienna approved the study (EK 16-194-VK). All of the participants provided written informed consent before data collection, which was carried out between July 2016 and January 2017.

The participants were recruited through mail by (1) the Department of Rehabilitation Medicine, University Medical Centre Groningen, the Netherlands, (2) De Hoogstraat Rehabilitation Centre Utrecht, the Netherlands, and (3) Otto Bock Healthcare Products GmbH (OPHP), Vienna, Austria. Prosthesis users were selected based on their medical files. Inclusion criteria were adults with unilateral upper limb defects at the transhumeral, transradial, or wrist level and at least six months of experience with conventional control and/or pattern recognition control of myoelectric multi-function upper limb prostheses (Ottobock Michelangelo Hand or Touch Bionics i-limb, which is now Touch Bionics by Össur) or RSL Steeper Bebionic (which is now Ottobock Bebionic). The inclusion criteria for the therapists were at least six months of experience treating prosthesis users who used one of these prosthetic hands.

Qualitative, semi-structured interviews were chosen to achieve in-depth understanding of users’ and therapists’ opinions on multi-function prostheses. The interview guide was developed in English prior to conducting the first interview and was not altered during the study (see supporting information S1 Text). It was then translated into German and Dutch by native speakers. All of the interviews were conducted by a facilitator whose native language matched the participants’ native tongues. Dutch interviews were conducted by a medical student who was experienced with patient contact and taking patients’ histories. The first author was present at all of the interviews and intervened if necessary. Three German interviews were conducted by the first author, who is a native German speaker. Two remaining German participants were interviewed by author #5 and author #6, who are both native German speakers and attended two prior interviews in the presence of the first author. Both were experienced with the topic and patient contact. Author #5 knew these two participants and another third participant from earlier research, but other than that no relationship was established between the interviewers and any of the participants before the interviews.

All of the interviewers introduced themselves at the beginning of each interview, explaining their role and the goal of the study. Subsequently, demographics and general anamnesis were noted before open questions were asked about prosthesis control and use. The interviews were recorded with a smartphone and field notes were taken. The participants were told that they would receive a transcribed version of the interview that allowed them to make changes in case they felt that the transcribed version did not properly reflect their opinions. Each interview lasted between 25 and 45 minutes, and no repeat interviews were conducted.

Given the heterogeneity of the target group (e.g., age, gender, prosthesis type, control type, and user/therapist), we estimated a required sample size of 15–20 participants to reach data saturation and contacted an initial sample of 16 users and 7 therapists. Fifteen users and seven therapists provided written informed consent to participate. We conducted a first round of interviews with this initial sample and decided to discuss further recruitment of participants depending on whether data saturation was reached within this sample. Data saturation was reached if the last three interviews of the initial sample did not reveal new themes, i.e. if all of the last three interviews could be completely coded with themes which had emerged from prior interviews.

The study data were reported based on the consolidated criteria for reporting qualitative research (COREQ).[19] The data were analysed according to the thematic framework approach.[20] All of the Dutch interviews were transcribed by an assistant (a student of physiotherapy and native Dutch speaker) who was not present during the interviews. All of the German interviews were transcribed by the first author. The transcribed documents were sent to the participants for corrections. Two interviews were returned with changes mostly related to syntax corrections. The overall quality of the recordings was excellent, with less than 1% of audio data that could not be understood. These passages were marked in the transcription. A 5-step framework approach[20,21] was used for the data analysis:

1. Familiarisation: The first and the last author read all of the interviews independently and created an initial main theme set based on the interview data and in line with the main goals of the study. Both of the authors met to discuss their theme set until a consensus was reached for a main theme set.
2. Identifying a thematic framework: After reading a first prosthesis user interview, the first and the last author independently created a thematic framework with subthemes based on the initial main theme set and coded the interview data accordingly using qualitative data analysis software (Atlas.ti version 7.5.16, Atlas.ti GmbH, Berlin, Germany). Comparing those two frameworks revealed no major differences. After agreeing on a merged version, both of the authors continued to read the remaining interviews and new subthemes and amendments to the framework were discussed in face-to-face sessions until a consensus was reached. As both of the authors agreed that no new themes emerged from the last three interviews, data saturation was reached and no further participants were recruited. The final themes and subthemes are shown in Table 1.
3. Indexing: The first author coded all of the interview data using Atlas.ti.
4. Charting: Quotes were sorted by themes and subthemes in Atlas.ti.
5. Mapping and interpretation: The first and the last author read the charted quotes and wrote summarised interpretations of all of the quotes according to each subtheme. Both of the authors repeatedly met to discuss and contrast interpretations for each subtheme until a consensus was reached.

**Table 1:** Main themes and subthemes of thematic framework, and count of how many times themes were mentioned.

Theme / Subtheme	Total count themes were mentioned	Number of prosthesis users who mentioned theme (total number prosthesis users: 15)	Number of therapists who mentioned theme (total number of therapists: 7)
**Control**	** **		
2-Site Control: General Remarks	21	7	5
2-Site Control: Positive Remarks	19	9	1
2-Site Control: Negative Remarks	121	14	6
Pattern Recognition Control: Advantages*	47	4	1
Pattern Recognition Control: Disadvantages*	24	4	1
User Wishes	24	7	2
**Prosthesis**			
Positive Remarks	37	10	6
Negative Remarks	106	13	6
Expectations	34	9	5
Integration, Acceptance, Learning	90	14	7
Reasons for Wearing / Using	68	14	6
**Activities**			
Activities generally mentioned	255	15	7
Important Activities	97	13	7
Problematic Activities	73	13	6

*Note: Only four prosthesis users and one therapist experienced with pattern recognition were included in this study.

## Results

Fifteen users and seven therapists participated in this study (Table 2). Four of the users and one therapist were experienced with pattern recognition control. All prosthesis users had experience with a standard one-degree-of-freedom myoelectric prosthesis. They had either used a standard prosthesis before being fitted with a multi-function prosthesis or they used both prostheses alternately. Since the data from the users and therapists were largely congruent, results are reported for both groups under the term “participants” unless distinctions are made explicitly in the text (“users” versus “therapists”). The numbering of the participants’ synonyms (e.g., “P1” or “T2”) does not correlate with the order of the entries in Table 2. Further details are available from the authors.

**Table 2:** Participant’s characteristics.

**Users**										
Sex	Age	Cause of Deficiency	Affected Side	Hand Dominance Before Amputation	Level of Deficiency	Prosthesis	Experience with multi-function prostheses (years)	Average Prosthesis Use (hours/day)	Comorbidities	Experience with Pattern Recognition
M	57	Trauma	L	R	Wrist Disarticulation	bebionic	1	12 to 14	/	/
M	28	Congenital	R	/	Trans Radial	i-limb revolution	1,5	3	/	/
F	22	Trauma	R	R	Trans Radial	i-limb revolution	2,5	5	/	/
M	46	Complex regional pain syndrome	L	R	Trans Radial	i-limb quantum	1,5	4	/	/
M	53	Trauma	L	L	Trans Radial	i-limb quantum	8	8 to 12	/	/
M	57	Trauma	R	R	Trans Radial	Michelangelo	3	16 to 17	Diabetes Type II	/
F	48	Complex regional pain syndrome	R	R	Trans Radial	i-limb revolution	2	14	Lyme disease	/
M	53	Complex regional pain syndrome	R	R	Trans Radial	bebionic	2	10	/	/
M	39	Trauma	R	R	Trans Radial	Michelangelo	0,5	10 to 13	/	/
M	61	Infectious disease	R	R	Wrist Disarticulation	bebionic	2	10	Leukemia	/
M	60	Congenital	L	/	Trans Radial	bebionic	1,5	14 to 16	/	/
M	29	Trauma	R	R	Trans Radial	Michelangelo	3,6	3 to 14	/	yes
M	32	Trauma	R	R	Trans Radial	Michelangelo	6	14	/	yes
M	30	Trauma	L	L	Trans Radial	Michelangelo	1	10 to 12	/	yes
M	41	Trauma	R	R	Trans Radial	Michelangelo	6	12 to 14	/	yes
**Therapists**							** **			
Sex	Age	Experience with multi-function prostheses (Prosthesis Type)			Experience with multi-function prostheses (Time, years)					
F	62	bebionic			0,5					
F	59	i-limb, bebionic, Michelangelo			7					
F	43	i-limb, bebionic, Michelangelo			9					
F	35	i-limb, bebionic, Michelangelo			9					
M	32	i-limb, bebionic, Michelangelo			9					
F	40	i-limb, bebionic, Michelangelo			8					
F	38	Michelangelo			4					

An overview of the interview themes and how many times they were mentioned by how many participants is given in Table 1.

### Conventional control in multi-function prostheses

Seven of the 15 users said they were satisfied with conventional control, whereas five users clearly reported dissatisfaction and three users made ambiguous statements about satisfaction. The therapists confirmed that satisfaction differed substantially among the users. Nearly all of the participants described the process of switching between prosthesis functions as a major problem of conventional control in multi-function prostheses. Producing “mode switching” muscle contractions (such as co-contraction of muscles) that are required for function switching in conventional control was described as too time consuming, unreliable, non-intuitive, and mentally exhausting. Some users reported difficulties in producing the required muscular contractions.

*“The control… it always takes effort […] And if it takes me a lot of effort to do a certain muscle contraction for a certain task to perform*. . . . *Then I do it once*, *then I do it twice*, *but I won’t do it a third time*.*”* (P2)

*“It is more that you sometimes think* … *this switching to another grip function* … *That you have to use a trigger* [i.e., the muscular contraction that activates the switching] *and then you continue with the next step*. *Then it takes you a bit longer* … *If you are holding something in your hands then it’s indeed annoying*.*”* (P12)

*“*…*my third trigger signal was that I* … *that I have to move both muscles at the same time* [co-contraction], *but I just don’t get that* … *I don’t get that under control*. *It’s just very difficult*.*”* (P11)

*“Well*, *you just take the path of least resistance*. *And I won’t slow down my movements just to utilise my prosthesis*.*”* (P16)

Instead of adjusting the prosthesis to the given tasks by switching the prosthesis function, many users expressed their preference for using compensation strategies (e.g., trunk or shoulder motions) to appropriately position the prosthesis. Many of the users also indicated that for tasks that can be completed unimanually, they would rather use their sound hand because the task could be done much quicker and with less effort. These findings were largely confirmed by the therapists. The data also showed that most of the users did not exploit more than two or three prosthesis functions because more functions and their corresponding “mode switching” muscle contractions were perceived as confusing and hard to remember.

*“I only use two grip modes now because the rest* [of the functions] *I can choose from… they are just…*. *they just remain difficult*.*”* (P8)

*“Actually it* [the prosthesis] *should do it faster* … *it has to switch* [between functions] *faster instead of the user quickly adjusting his body position*.*” (T3)*

An exception was two users who utilised the gesture control feature of the i-limb quantum prosthesis. Based on inertia-measurement units, this feature allows the switching of prosthetic functions by moving the prosthesis in a specific pattern. Although both of the users felt that function switching with this method still required too much time, they appreciated the gesture control feature, as it was easier to control and felt more intuitive than the muscle contraction switch command signals. However, a third user who had tested different prostheses types and tried the gesture control was clearly not satisfied with this feature as he described it as impractical.

*“But yeah*, *the rest*, *yeah*, *perfect…*. *And then all the way left*, *right… switch to fine pinch… this way and then back and forth* [demonstrating gesture control]. *But… what I do find is that it all takes too long*.*”* (P4)

*“The gyroscope* [i.e., the gesture control feature] *in the i-limb is really nice*, *but in practise* … *that’s only my modest opinion* … *it’s an absurdity*. *Because if you walk you can’t do anything with it*, *you always have to stand still* … *Those are things which are* … *in practise* …*they’re just impractical*.*”* (P9)

### Pattern recognition control in multi-function prostheses

The users who were experienced with pattern recognition were all active users of conventional control. They had access to a custom-made, not commercially available, pattern recognition system provided by Ottobock Healthcare Products during several trials over the course of one to two years. All of these four participants used the OttoBock Michelangelo Hand with conventional control and during the pattern recognition try-outs. Sockets for this system were custom-made and individualized for each prosthesis user by the same prosthetist. The sockets were fitted with eight commercially available double differential electrodes (13E200 = 50 AC, Otto Bock Healthcare Products GmbH, Vienna, Austria). A generally well-known and often-used pattern recognition method was employed based on a linear discriminant analysis (LDA) classifier. This pattern recognition system is described in detail elsewhere.[22] The time of pattern recognition experience varied between the users according to their availability and ranged between 10 and 50 hours (accumulated and separated over several days). The therapist experienced with pattern recognition who was included in this study trained the four users during their trial phases.

All five of the participants (four users and one therapist) experienced with pattern recognition stated that if this control worked properly, it was clearly faster and felt more natural than conventional control.

*“If it works* [pattern recognition control], *it is clearly faster*.*”* (P14)

*“Well*, *the PatRec* [the pattern recognition control] *surely is* … *with regard to how the control feels… more like it was before with the* [intact] *hand*.*”* (P13)

The main reason for faster control and a more natural feeling was that pattern recognition allowed seamless transitions between prosthesis functions without switching commands. Some of the participants also stated that more functions of the prosthesis were exploited because the transition from one function to another was not perceived as a burden.

*“I noticed that… because I have a standard grip*, *the key grip …when I have the pattern recognition prosthesis*, *I also use the fine pinch grip much more frequently*, *because I do not have to switch* [via switching commands].*”* (P15)

However, all of the participants also explained that pattern recognition lacked robustness in real-life usage. Specifically, the movements of the socket with regard to the skin and pressure forces acting on the socket could produce signal artefacts that caused involuntary prosthesis movements. Moreover, control was described as unreliable in changing temperatures and humid conditions. The participants said that although robustness issues were also evident in conventional control, they were less pronounced than in pattern recognition control. Additionally, training was perceived as time-consuming and exhausting.

*“Since I do a lot of heavy work*, *e*.*g*. *carrying bags of potatoes*, *I often have a lot of weight hanging on my hand*, *and with the pattern recognition prosthesis this means control quickly becomes unreliable*.*”* (P13)

*“First of all*, *pattern recognition requires a lot of training before it works properly*.*”* (P14)

### Multi-function prostheses

The most prominent remarks about multi-function prostheses were that these devices were perceived as too fragile, and especially broken fingers and gloves were mentioned as recurring issues. Interestingly, out of six users who had no such complaints, five users had a Michelangelo Hand and one had a Bebionic Hand. When damage occurred, most of the participants stated that repairs usually take a long time due to slow supply from the manufacturers.

*“*[…] *but I am way too scared that something goes wrong*: *BAM*, *2000 euros* … *or the glove gets damaged*, *BAM*, *300 euros*. *Then I think* … *well*, *I rather won’t try it then*. […] *It’s just too expensive*. *And if you had it damaged a couple of times and then you can’t use your prosthesis for two or three weeks*…*”* (P4)

Moreover, a particular complaint of the male prosthesis users was that grip force was too weak to substantially support the sound hand in heavier bimanual tasks. When lifting heavy objects, the prosthetic hand was reported to open unintentionally. Due to this lack of force control and high chances of prosthesis damage, many users tried to avoid using their prosthesis for tasks that they perceived as too difficult. Nonetheless, some of the participants still saw a functional advantage in multi-function prostheses, because multi-articulating fingers close better around objects and can therefore provide a more reliable grip, e.g., when lifting a mug. Moreover, the possibility of choosing from different motor functions was appreciated by some of the prosthesis users and most of the therapists described the various grip modes as adding value.

*“With this one*, *I can grasp in a good*, *stable manner*. *For instance*, *holding your keys or a key card without objects slipping through your hand*. *That’s really nice*.*”* (P3)

*“There are a couple of grip modes that users can choose from and the lateral grip… the key grip is really of additional value in my opinion*.*”* (T6)

### Activities

The majority of the users stated that they used their multi-function prosthesis most often for tasks that cannot be executed unimanually, which was strongly confirmed by the therapists. During these activities, the sound hand played the leading and manipulating role while the prosthetic hand took a supporting role to stabilise the objects.

*“With my prosthesis*, *I grab everything that is… I don’t know how to express it… Everything that is static*. *What I can hold in one position*. *And with my other* [intact] *hand*, *I do everything that is dynamic*.*”* (P3)

*“Well*, *the leading hand is always the intact hand*, *and stabilisation and holding* [of objects] *is done by the prosthetic hand*.*”* (P16)

*“To cycle*, *to push a pram (difficult to keep on track with one hand)*, *to carry a tray*, *to carry a crate of beer… objects that are supposed to be lifted bimanually*, *to open a bottle while standing (you can’t use your legs to fixate the bottle then)*, *to fasten a belt*, *to put on your pants… those things are all possible with one hand only*, *but they’re difficult*.*”* (T7)

Specific activity domains varied strongly depending on the individual user. Highly recurring domains of the International Classification of Functioning, Disability, and Health (ICF) components Activities and Participation are shown in Table 3.

**Table 3:** Recurring domains of the International Classification of Functioning, Disability and Health (ICF) components “Activities and Participation”.

ICF Domains (Activities & Participation)	Exemplary Quotes
**Mobility** (Lifting/Carrying/Handling Objects, Driving (bike, car, motorbike)	“It supports my right side actually. So simply holding things, bigger things, lifting objects… That won’t work with my right hand alone” (P1)
	“Especially driving vehicles, the tractor, the car. . . . because of the gearshift”(P3)
**Self-Care** (Eating/Drinking/Dressing	“Just the daily things where you need a second hand: Open a jar, open a bag of chips, zip my coat, tie my laces…” (P5)
	“For instance, if you are are eating from a barbecue. Yeah I always needed to go to a table to put my plate down there. With my prosthesis I can stand, hold my plate and just eat.”(P14)
**Domestic Life** Preparing Meals/ Doing Housework	“Yeah for example to iron, that wont work with one hand only. Or for example to cut food”(P2)
	“A bit of leisure time activities, although that’s pretty diverse. This could be be tinkering for example, we hear that a lot.” (T5)

## Discussion

The aim of this study was to capture the users’ and therapists’ opinions on two recent technological advancements in upper limb prosthetics: multi-function prosthetic hands and pattern recognition control. The results suggest that both technologies offer potential benefits for users and indeed match their desires expressed. However, both technologies appear to have serious drawbacks that limit their functionality.

Myoelectric multi-function upper limb prostheses offer a variety of functions (such as grip and wrist movements). The desire for such functions has been repeatedly expressed by prosthesis users.[6] Indeed, the results of this study partly confirm earlier findings, wherein users reported that multi-function prosthetic hands facilitate easier and more reliable grasping of objects, mainly due to multi-articulating fingers.[10,13,14] On the other hand, our results indicate that under conventional myoelectric control, the benefits of multiple functions (e.g., wrist rotation and multiple grip types) appear to be strongly limited because switching between these functions cannot be executed quickly and intuitively. In addition to difficulties with function switching under conventional control, poor mechanical robustness was another barrier frequently encountered in multi-function prostheses, although our data suggests that these issues are less pronounced in the Michelangelo hand, compared to the Bebionic hand and the iLIMB hand.

Following the path of least resistance, many users thus avoided engaging their prosthetic hand due to difficulties with control and fear of damaging the prosthesis. These findings confirm earlier reports of one-handed behaviour in unilateral upper limb prosthesis users.[11,23] The current study design does not allow a quantification of prosthesis use in daily tasks, but the results show that state-of-the-art multi-function prostheses remain simple stabilising and supporting tools for activities of daily living requiring two hands.

The limitations of conventional control might be overcome by pattern recognition control. This study’s results indeed confirmed that machine learning algorithms have the potential to facilitate intuitive and rapid prosthesis control. This is in line with users’ desire for more dexterous prosthesis movements.[6,24] However, pattern recognition control was reported to be unreliable in many instances. Most importantly, control reliability was reported to be at risk in situations that are seldom reflected in lab conditions. Changes in temperature and air humidity or external forces acting on the socket (e.g., due to weight hanging on the prosthesis) were stated to quickly turn prosthesis control with pattern recognition from manageable to difficult or impossible. It is questionable whether satisfaction will improve when problems with non-intuitive conventional control are replaced by intuitive but unreliable control.

It is also noteworthy that all of the participants experienced with pattern recognition stated that this type of control required extensive training before it works properly. Similar results were found by Resnik et al.[17,18], although their study was based on a different pattern recognition system and another type of prosthetic hand/arm.

This study’s strength is a qualitative approach that allowed a relatively large sample of users and therapists to express their opinions on state-of-the-art prosthetic technologies. Nonetheless, this study has limitations.

First, some of the interviews were conducted by phone while others were administered via face-to-face sessions, which might have affected the quality of the data. However, Novick (2008) argued that it is unwarranted to favour any particular interview mode for qualitative interviews. The data from the telephone interviews seem to be comparable to the data from the in-person interviews.[25]

Second, the interviews were conducted in languages other than English, while the findings are presented in English. This might have introduced challenges into the analysis process.[26] During the analysis, we therefore maintained the original language as long as possible and translated the quotes into English only during the final step of writing the manuscript.[26]

Third, ideally this study would have been conducted on a more homogenous sample of prosthesis users (e.g. users with the same type of prosthetic hand) to draw conclusions specific for this sample. However, due to the sparsity of users who are experienced with such devices, this was not feasible. In spite of that, the data showed no evident differences for different prosthetic hands, with the exception that the Michelangelo Hand was less frequently described as too fragile, compared to the other two prosthetic hands.

Finally, the pattern recognition users’ experiences could not be quantified in a more detailed way. Findings regarding pattern recognition should therefore be interpreted with caution, especially given the small sample of experienced pattern recognition users. Moreover, these findings might be specific for the type of users (all male and all trans-radial amputations) and the type of pattern recognition control that the participants used which was not a commercially available system. Nonetheless, the main findings regarding extensive training and control robustness issues in pattern recognition control are in agreement with other studies.[17,18]

Moreover, findings with regard to control robustness might be influenced by the skin-electrode interface, as pattern recognition control has been shown to be sensitive to non-stationarities such as electrode shift or changing skin conditions.[27,28]

This study found that irrespective of the available grip and wrist functions, multi-function prosthetic hands are unlikely to be actively used if the risk of damage is exceptionally high or if the control is too difficult. For individuals with unilateral upper limb defects, current state-of-the-art multi-function prosthetics remain a stabilising and supporting tool for bimanual tasks, where active grasping and the manipulation of objects is seldom performed with the prosthesis. This might contribute to numerous musculoskeletal complaints (e.g., in the non-affected limb and upper back/neck level) in prosthetic users.[29] Moreover, the chances of device embodiment are presumably low if the user cannot rely on easy and consistent control or on the mechanical robustness of the prosthesis.[30] These factors could influence device abandonment risk.[1,31] In summary, the potential benefits of multi-function prosthetic hands cannot be optimally utilized under conventional 2-site control.

Pattern recognition has the potential to become a major advance in multi-function prosthesis control and thus might be an important step in improving functionality and acceptability of upper limb prosthetics. However, for future assessments of pattern recognition control, it is important to evaluate the control under conditions that challenge the robustness of the system and resemble realistic usage, since the subjects of this study described the control reliability as the main obstacle to pattern recognition control. Likewise, it should be evaluated whether improved skin-electrode interfaces such as electrode-liners will improve the control robustness.[32] Moreover, when evaluating pattern recognition control and its advantages over conventional control, it should be taken into account that extensive training might be required before potential benefits are apparent. For future studies, we consider it important to assess whether the latest developments in commercially available pattern recognition systems (i.e. the second iteration of *COAPT complete control* and *OttoBock Myoplus*) demonstrate improvements in required training times and control robustness.

## Supporting information

S1 Text Interview guide. (DOCX)
