# Supplementary material for: Users’ and therapists’ perceptions of myoelectric multi-function upper limb prostheses with conventional and pattern recognition control
Source: PLoS One. 2019 Aug 29;14(8):e0220899. doi: 10.1371/journal.pone.0220899 (PMC6715185; doi:10.1371/journal.pone.0220899)
Supplement: S1 Text — (DOCX) [file pone.0220899.s001.docx]

**S1 Text: Interview Guide**

(Questions for users experienced with pattern recognition control are marked with *. The interview guide for therapists consisted of the same questions as shown here, only altered to match the therapists’ perspective (e.g. (1) “Could you identify the main reasons *for a user* to wear a prosthesis?”

(1) Could you identify the main reason to wear a prosthesis?

- Cosmetic vs. Functional?

- If Functional: For which domains of activities of daily living

(2) How satisfied are you with the overall functioning?

*-Pattern recognition: Preferred over conventional?

(3) With regard to the “mode switching” trigger signals which are needed to switch between prosthesis (grip-) mode with conventional control, can you give a rough estimation how often you use these triggers per day?

- Do you see advantages/disadvantages of these triggers?

- Given that with pattern recognition control trigger signals aren’t necessary, can you imagine a situation where this would have an effect?

*-Pattern recognition: Where do you see the main difference between conventional and PR control?

(4) With regard to activities of daily living, are there any tasks that you would describe as very important for you (e.g. tasks which you perform often, or tasks which are crucial)?

- What is the prosthesis needed for in these tasks?

*- Pattern recognition: Do you use your prosthesis differently with pattern recognition in these tasks?

(5) Can you think of specific tasks which you can only perform wearing your prosthesis?

- Which parts are performed with the prosthesis (and what is done by the sound hand)?

*- Pattern recognition: Do you use your prosthesis differently with pattern recognition in these tasks?

(6) Are there any tasks which you encountered as very difficult or impossible to perform with your prosthesis?

-What is it exactly that makes these tasks difficult?

*- Pattern recognition: Did pattern recognition alter these problems?

(7) Are there any activities which require an awkward body positioning?

(8) Are there tasks which you accepted as not doable due to the prosthesis?

*- Pattern recognition: Did pattern recognition have an effect on this?

(9) Are there tasks which you would describe as well doable, meaning you are satisfied with the performance of the prosthesis?
